# Lymph-Targeted Resveratrol-NLCs Improve Oral Bioavailability: Validation via Rat Mesenteric Lymph Collection System and In Vivo Safety

**DOI:** 10.3390/ani16152337

**Published:** 2026-07-31

**Authors:** Xiaorui Zhang, Wenli Shi, Xinlin Yang, Yuchen Lin, Bo Yang, Hui Deng, Daojin Yu, Shuaizhen Zhou

**Affiliations:** 1 Fujian Key Laboratory of Traditional Chinese Veterinary Medicine and Animal Health, College of Animal Sciences, Fujian Agriculture and Forestry University, Fuzhou 350002, China; xiaorui.zhang@sibcb.ac.cn (X.Z.); swli795@163.com (W.S.); 13774852102@163.com (X.Y.); 18905991017@163.com (Y.L.); ybvet@fafu.edu.cn (B.Y.); deng12345hui@163.com (H.D.); 2 Core Facility of Molecular Biology, Center for Excellence in Molecular Cell Science, Shanghai Institute of Biochemistry and Cell Biology, Chinese Academy of Sciences, Shanghai 200031, China

**Keywords:** resveratrol, RES-NLCs, intestinal lymphatic transport, oral bioavailability, pharmacokinetics, mesenteric lymph duct

## Abstract

Resveratrol (RES) is a natural polyphenolic compound characterized by poor aqueous solubility and significant first-pass metabolism, resulting in extremely low oral bioavailability. Although resveratrol-loaded nanostructured lipid carriers (RES-NLCs) have shown potential in enhancing oral absorption, direct experimental evidence for their intestinal lymphatic transport mechanism remains limited, and existing explanations is largely based on indirect inference. RES-NLCs were prepared, and their pharmacokinetics and lymphatic transport characteristics were evaluated using a laboratory-established mesenteric lymph duct–jugular vein assisted reflux model in rats. Simultaneously, a 28-day repeated-dose toxicity study was conducted in ICR mice. Pharmacokinetic results showed that compared with RES-Sol, RES-NLCs increased Cmax by approximately 2.3-fold, improved relative bioavailability by 7-fold, and achieved an absolute bioavailability of 176%. The lymphatic transport model confirmed that RES-NLCs are absorbed via the intestinal lymphatic pathway. In the 28-day repeated-dose toxicity study, no mortality or obvious clinical symptoms were observed at a dose of 10 mg/kg. The RES-NLCs group exhibited increased liver coefficient and decreased spleen coefficient. Hematological analysis showed a mild increase in red blood cell count, along with decreases in mean corpuscular volume and red blood cell distribution width coefficient of variation. Serum biochemistry revealed significant elevations in aspartate aminotransferase and alanine aminotransferase (*p* = 9 × 10^−5^ and *p* = 4.02 × 10^−7^, respectively). However, no significant differences were observed in the organ coefficients of the heart, lungs, kidneys, or brain. Body composition and magnetic resonance imaging showed no abnormalities, and histopathological examination of major organs including the liver, stomach, and intestines revealed no structural damage. These findings provide direct evidence that RES-NLCs enhance the oral bioavailability by promoting intestinal lymphatic uptake, suggesting that this system may serve as an effective delivery platform for poorly soluble hydrophobic drugs.

## Simple Summary

Resveratrol is a plant-derived natural compound present in grapes, peanuts, and various other plants. It has antioxidant and anti-inflammatory benefits, but it is very poorly absorbed by animals or humans. In this study, we made resveratrol into tiny lipid particles and gave it to rats by mouth. The results showed that these particles increased the amount of resveratrol in the blood by 7 times. First, we established a rat model for collecting intestinal lymph. Unlike traditional methods, this system enables continuous lymph/blood collection and dosing in conscious, freely feeding rats, avoiding interference from surgical stress, anesthesia, and restraint. We also proved that the particles travel through the gut lymph vessels directly into the bloodstream, bypassing the liver where the drug would otherwise be broken down. This explains the much better absorption. We then gave the particles to mice every day for 28 days. No deaths or obvious side effects were seen. Some liver-related measures in the blood went up, but the liver, intestines, and other major organs looked completely normal under the microscope. These findings suggest preliminary tolerability, although the elevated liver-related biochemical markers require further investigation. This research provides a basis for developing safe and effective veterinary products from natural plant compounds. It could help improve the health of farm animals while reducing the need for antibiotics.

## 1. Introduction

As livestock and poultry production becomes increasingly intensive and large-scale, animal performance and herd health face growing challenges. On the one hand, bacterial resistance and drug residues resulting from the long-term or irrational use of antibiotics have become major threats to food safety and public health, creating an urgent need for safe and effective antibiotic alternatives [1,2]. On the other hand, oxidative stress, immune suppression, and metabolic disorders commonly observed in modern breeding environments directly compromise animal growth efficiency, reproductive performance, and product quality [3]. Against this backdrop, natural plant-derived compounds, which offer multiple bioactivities and excellent safety profiles, have emerged as a central focus in the development of novel functional feed additives and green veterinary drugs. Their multitarget effects, low residue, and minimal toxicity make them promising candidates for sustainable livestock production [4].

Resveratrol (RES), a naturally occurring non-flavonoid polyphenolic compound [5], has garnered considerable attention in the veterinary field due to its anti-inflammatory [6], antioxidant [7], immunomodulatory [8], and lipid metabolism-regulating properties [9]. Studies have shown that RES may enhance animal growth performance, meat quality, and reproductive efficiency by improving gut health, increasing stress resistance, and modulating nutrient metabolism [10]. For instance, in ruminant production, RES has demonstrated potential benefits in improving rumen fermentation efficiency and reducing methane emissions [11]. In farm animal production, RES is therefore considered a potential functional dietary compound for alleviating oxidative stress, immune suppression, and metabolic disturbances associated with intensive rearing conditions. However, its practical use in feed or veterinary formulations is limited by poor water solubility, chemical instability, along with a pronounced first-pass effect after oral administration and extremely low bioavailability. These drawbacks severely limit the ability of RES to reach the target sites and maintain effective therapeutic concentrations, thereby hindering its practical application [5]. Therefore, improving the oral absorption of RES is essential for its further development as a functional feed additive or veterinary formulation.

Nanostructured lipid carriers (NLCs), as an advanced nano-delivery system, can significantly enhance the solubility and stability of hydrophobic drugs and improve oral bioavailability by promoting intestinal absorption and lymphatic transport [12]. Thus, encapsulating RES into NLCs represents a promising strategy to achieve effective delivery and fully realize its pharmacological potential. Nevertheless, any new formulation designed to alter the in vivo behavior of a drug—particularly nanocarriers—introduces dual challenges. On one hand, nanocarriers may alter the drug’s release kinetics, biodistribution, and elimination profile [13]; on the other hand, the physicochemical properties of the carrier materials themselves (e.g., particle size, surface charge, composition) can lead to unintended accumulation in specific tissues or organs, thereby posing new safety concerns not observed with the free drug [14].

Although current research on RES-NLCs has made considerable progress in formulation optimization, physicochemical characterization, and short-term efficacy evaluation, systematic and long-term in vivo safety assessments of this nanoformulation remain severely lacking. This “efficacy-focused, safety-neglected” evaluation approach leaves a significant gap and fails to provide adequate support for the further development of nanoscale veterinary formulations. Notably, the biosafety of nanocarriers themselves warrants particular attention. Using a chick embryo model, Zhang et al. found that blank nanocarriers stabilized with different materials (e.g., Tween 80 and carboxymethyl chitosan) induced higher mortality, teratogenicity, and even genotoxicity than the drug-loaded nanoparticles [15].

Therefore, based on our research group’s previously established nanostructured lipid carrier platform [16], we prepared RES-NLCs and evaluated their pharmacokinetic characteristics and lymphatic transport following oral administration in real time using a conscious, freely fed rat model with mesenteric lymph duct–jugular vein cannulation. Additionally, a 28-day repeated-dose toxicity study was conducted in ICR mice to comprehensively evaluate the subchronic toxicity of RES-NLCs and preliminarily explore the potential relationship between the observed toxic effects and the properties of the nanoformulation. The findings of this study will provide fundamental pharmacokinetic and safety data to support the application of RES-NLCs as a potential veterinary formulation, and will also offer experimental evidence for the safety evaluation of nanoscale veterinary drugs.

## 2. Materials and Methods

### 2.1. Experimental Animals

SPF ICR mice were obtained from Zhejiang Vital River Laboratory Animal Technology Co., Ltd. [SYXK (Shanghai) 2023-0013] (Jiaxing, China), with quality certificate No. 20260305Abzz06190000442. SPF SD rats were purchased from Shanghai SLAC Laboratory Animal Co., Ltd. [SCXK (Shanghai) 2022-0004] (Shanghai, China), with quality certificate No. 20220004095958. All animals were housed in individually ventilated cages (IVC) within an animal barrier facility, with free access to water and food. Environmental conditions were controlled at 23 ± 2 °C, with a relative humidity of 40–70%, noise ≤ 60 dB, and illumination of 20 lx under a 12 h light/12 h dark photoperiod. The animal housing density was 3 rats per cage and 5 mice per cage. All animals were acclimatized for 7 days prior to the experiment. All experimental procedures in this study were reviewed and authorized by the Institutional Animal Care (No. SIBCB-S119340-2112-045) and Use Committee and were performed under aseptic conditions in accordance with the Guide for the Care and Use of Laboratory Animals (National Academies Press, 2011). Sample sizes were selected based on previous experience with the mesenteric lymph duct–jugular vein catheterization model, preliminary pharmacokinetic observations, technical feasibility, and the 3Rs principle to reduce unnecessary animal use.

### 2.2. Drugs, Reagents, and Main Instruments

Resveratrol was purchased from Titan Technology (Shanghai) Co., Ltd., China (Shanghai, China). Glyceryl stearate (CAS No. 123-94-4), glyceryl monooleate (CAS No. 111-03-5), and phosphatidylcholine (CAS No. 51779-95-4) were purchased from TCI (Shanghai) Development Co., Ltd., China (Shanghai, China). An ultrasonic cell disruptor (JY92-IIN) was obtained from Xinzhi Biotechnology Co., Ltd., China (Ningbo, China). A liquid chromatography–tandem mass spectrometry system (QTRAP 6500+) was purchased from Sciex, Marlborough, MA, USA. An animal body composition and MRI analyzer (NM42-060H-1) was purchased from Niumag Analytical Instrument Corporation, Suzhou, China. An automatic biochemical analyzer (4600) was purchased from QuidelOrtho, San Diego, CA, USA. An automatic modular animal blood and body fluid analyzer (XN-1000V) was purchased from Sysmex Corporation, Kobe, Japan. A small animal ultrasound diagnostic system was purchased from FUJIFILM VisualSonics, Tokyo, Japan. The rat 2-channel VAB tether (VABR2T/25), rat 2-channel vascular access button (VABR2B/25R22), rat 2-channel VAB loop connector (VABR2L), PinPort injector (PNP3M), 2-channel swivel (375/D/25LT), multi-axis counterbalanced arm (MCLA/MED), rat sampling cage apparatus (MTANK/WF), and polyurethane (PU) catheters (BTPU-027, BTPU-040) were all purchased from Instech, Plymouth Meeting, PA, USA.

### 2.3. Formulation Preparation

#### 2.3.1. Preparation of RES-NLCs

RES-NLCs were prepared using the emulsion-ultrasonication method based on the lipid carrier platform previously established by our research group, with RES used as the model drug [16]. Briefly, RES (10 mg), H125 solid–liquid lipid mixture (400 mg; consisting of 200 mg glyceryl stearate, 100 mg glyceryl monooleate, and 100 mg phosphatidylcholine), and Tween 80 (200 mg) were dissolved in 2 mL of anhydrous methanol. The mixture was maintained in a 75 °C water bath and stirred using a thermostatic magnetic stirrer until the methanol was completely evaporated and no visible bubbles remained, resulting in the formation of a uniform lipid film. Subsequently, 9.4 mL of deionized water preheated to 75 °C was added as the aqueous phase, and the mixture was continuously stirred for 5 min to form a primary emulsion. The primary emulsion was then transferred to an ultrasonic probe processor precooled in an ice bath and subjected to intermittent probe sonication at 200 W with a pulse mode of 2 s on and 1 s off for 3 min. Finally, the resulting emulsion was kept in an ice bath for 1 h to allow solidification of the lipid nanoparticles, yielding RES-NLCs. Blank-NLCs were prepared using the same procedure except that RES was omitted. The final RES concentration in the RES-NLC dispersion was approximately 1 mg/mL.

#### 2.3.2. Preparation of RES-Sol

The resveratrol solution (RES-Sol) was prepared as follows: 10 mg of RES raw material was accurately weighed and placed in a small beaker, followed by the addition of 1 mL of anhydrous ethanol and 0.5 mL of Tween 80. The mixture was stirred magnetically until the drug was completely dissolved. The resulting solution was diluted with physiological saline to a final concentration of 1 mg/mL prior to use.

### 2.4. Pharmacokinetic Analysis of RES-NLCs

#### 2.4.1. Quantitative Analysis Method for RES

The LC-MS/MS quantitative method for RES was established with reference to previously reported LC-MS/MS methods for the determination of resveratrol in biological matrices [17,18,19]. Chromatographic conditions: An ACQUITY UPLC BEH C18 VanGuard pre-column (Waters, Milford, MA, USA, 2.1 mm × 5 mm, 1.7 µm) was used. The flow rate was set at 0.3 mL/min, the column temperature was maintained at 40 °C, and the injection volume was 2 µL. The mobile phase consisted of acetonitrile containing 0.1% formic acid and 0.1% formic acid in water (5:95, *v*/*v*). Detection was performed in multiple reaction monitoring (MRM) mode, and the quantitative ion pair was *m*/*z* 227.0 → 185.1. Calibration curves were established using blank plasma spiked with RES standard solutions over the concentration range used for pharmacokinetic sample analysis. Linearity was evaluated using weighted linear regression, and calibration curves were prepared with each analytical batch. The back-calculated concentrations of calibration standards were examined to support the reliability of RES quantification during the formal sample analysis. Representative chromatograms were also used to assess whether obvious endogenous matrix interference was present at the retention time of RES. The detailed LC-MS/MS calibration curve data and representative chromatograms are provided in Supplementary File S1. This LC-MS/MS method was used as a comparative quantitative method for preclinical pharmacokinetic analysis under identical analytical conditions among experimental groups.

Sample preparation: An aliquot of 60 µL of blank plasma sample was placed on ice, and 1.2 µL of standard solution at various concentrations was added, ensuring that the pipette tip was inserted below the liquid surface for accuracy. Subsequently, 240 µL of acetonitrile/methanol (1:1, *v*/*v*) was added. The mixture was vortexed for at least 20 s to ensure thorough mixing and then stored at −20 °C for 30 min. Afterward, the sample was centrifuged at 12,000× *g* for 15 min at 4 °C. The supernatant (100 µL) was transferred to an autosampler vial under a fume hood while the samples were kept on ice. All samples were processed under the same extraction, centrifugation, and injection conditions to ensure comparability between the RES-Sol and RES-NLCs groups.

#### 2.4.2. Establishment of a Mesenteric Lymphatic Duct–Jugular Vein Assisted Reflux Model in SD Rats

**Figure 1:**
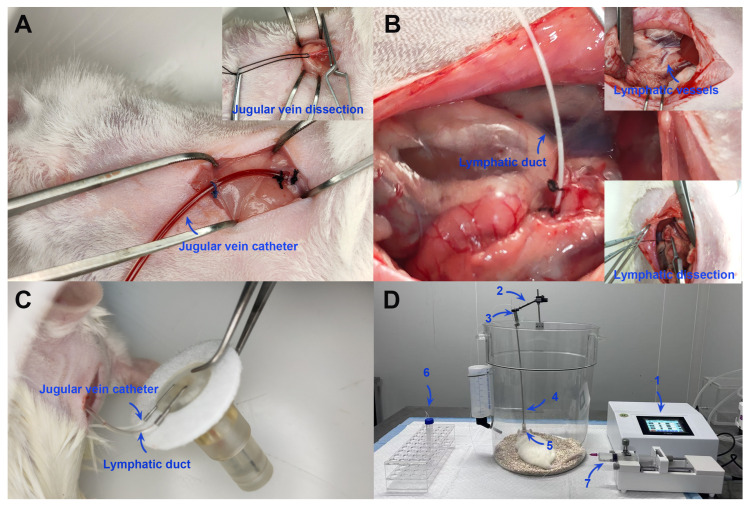
Physical image of the rat mesenteric lymphatic duct–jugular vein assisted reflux model; (**A**) Jugular vein catheterization. The external jugular vein was exposed and dissected, and a catheter was inserted into the jugular vein for venous access. The inset shows the jugular vein dissection. (**B**) Mesenteric lymph duct catheterization. The mesenteric lymphatic duct was identified, dissected, and cannulated under visual guidance. The insets show the exposed lymphatic vessels and lymphatic dissection. (**C**) Postoperative connection of the jugular vein catheter and mesenteric lymphatic duct catheter to the vascular access button (VAB). (**D**) Conscious lymph and blood sampling apparatus after model establishment. The numbered components indicate: 1, micro-infusion pump; 2, counterbalance arm; 3, 2-channel swivel; 4, VAB tether; 5, VAB; 6, lymph fluid collection tube; 7, Ringer’s solution.

Rats were used for pharmacokinetic and lymphatic transport studies because their larger vessel and lymphatic duct size facilitates catheterization, continuous lymph collection, and repeated blood sampling. Rats were anesthetized with Zoletil 50 at a dose of 50 mg/kg by intramuscular injection, and anesthesia was maintained with isoflurane inhalation delivered in oxygen at a flow rate of 0.5–1.0 L/min during surgery. The depth of anesthesia was adjusted according to the respiratory pattern, reflex responses, and general physiological status of the animals during the procedure. After observing decreased respiratory rate, muscle relaxation, and absence of pain reflexes, the rat was fixed on the operating table. Prior to surgery, the abdominal hair was shaved and the area was disinfected with 75% ethanol. An incision was then created along the abdominal midline in the upper two-thirds region to access the abdominal cavity. After exposure of the area around the left renal vein and inferior vena cava, the mesenteric lymphatic vessels adjacent to the mesenteric artery were carefully located. With the aid of a stereomicroscope, the mesenteric lymphatic vessel was dissected, and a 45° incision was made on the vessel wall. A lymphatic catheter was inserted to a depth of 1 cm, and both the catheter and the intestinal end of the lymphatic vessel were secured. The other end of the catheter was tunneled subcutaneously and connected to the lymphatic port of the vascular access button (VAB) positioned in the cervical region. Subsequently, the left neck vascular was exposed through a cervico-abdominal incision to identify the external jugular vein. A 45° incision was made on the vessel wall, and a venous catheter was inserted to a depth of 3.5 cm to reach the left subclavian vein. Both the catheter and the proximal end of the jugular vein were secured. The other end of the catheter was tunneled and connected to the jugular port of the VAB. After completing the cannulation, the VAB was connected to the VAB loop connector, the incisions were sutured, and the apparatus was connected to a 2-channel VAB tether, swivel, multi-axis counterbalanced arm, and sampling cage. The collection catheter was adjusted to the same height as the lymphatic cannula (Figure 1) [20].

The total surgical duration was approximately 60 min. During surgery, respiratory pattern, respiratory rate, respiratory amplitude, and pupil changes were continuously monitored. After surgery, rats were placed in a right lateral position on a 37 °C heating pad until full recovery of consciousness. Artificial tears were applied when necessary to prevent corneal drying, and gentle lower-abdominal pressure was applied when assistance with urination was required. Postoperative analgesia was provided with flunixin meglumine at 2 mg/kg by intramuscular injection every 12 h for 3 days. Enrofloxacin was administered at 2.5 mg/kg by intramuscular injection once daily for 3 days to prevent postoperative infection. When signs of dehydration were observed, lactated Ringer’s solution was administered subcutaneously at 5 mL/kg. Catheters were regularly flushed with heparinized saline during the first 3 postoperative days and locked with heparinized glucose solution to maintain patency.

After recovery, rats were housed individually with corn-cob bedding, and high-protein feed and water gel were placed on the cage floor to facilitate free access to food and water. Animals were monitored daily for abnormal posture, reduced appetite, depression, dehydration, abnormal body temperature, wound complications, catheter-related complications, and sustained body weight loss. Humane endpoints included severe distress, inability to eat or drink, persistent dehydration, marked body weight loss, severe infection or wound dehiscence, catheter-related complications causing distress, or pain not relieved by analgesic treatment. Animals reaching humane endpoints were euthanized immediately. The model was considered successfully established when the rat fully recovered from anesthesia, showed no severe postoperative distress, and both the jugular vein and mesenteric lymphatic catheters remained patent without leakage, obstruction, or displacement. Successful models also required continuous lymph flow through the mesenteric lymphatic catheter under conscious conditions, successful connection to the VAB tether–swivel–counterbalanced arm system, and completion of lymph or blood sampling during the planned collection period.

#### 2.4.3. Pharmacokinetic Study in SD Rats

SD rats with successful single jugular vein catheterization were randomly assigned to two groups, namely the RES-Sol group and the RES-NLCs group, with six rats in each group. After 12 h of fasting, rats received RES-Sol or RES-NLCs by oral gavage at a dose of 5 mg/kg. In conscious rats, blood samples (50 μL each) were withdrawn through the jugular vein catheter at 0.033, 0.083, 0.25, 0.5, 1, 2, 4, 8, 12, and 24 h after administration. The samples were transferred into anticoagulant tubes containing heparin sodium. Plasma was obtained by centrifugation at 4000 rpm for 15 min at 4 °C.

Separately, rats in which the mesenteric lymphatic duct–jugular vein assisted reflux model had been successfully established were randomly allocated into the RES-Sol and the RES-NLCs group, with six rats per group. Following 12 h of fasting, the corresponding formulation was administered orally at 5 mg/kg. Under conscious conditions, lymph fluid was continuously collected over three periods: 0–4 h, 4–8 h, and 8–24 h post-administration. To compensate for fluid loss during lymph collection, an equal volume of Ringer’s solution was immediately infused through the jugular vein catheter.

### 2.5. Safety Evaluation Methods for RES-NLCs

#### 2.5.1. Dosing Regimen of RES-NLCs in Mice

A 28-day repeated-dose oral safety evaluation was performed in female ICR mice with reference to OECD Test Guideline 407 [21]. Thirty-two female ICR mice were randomly divided into four groups (*n* = 8 per group): NA group, Blank group, RES-Sol group, and RES-NLCs group. Mice in the RES-Sol group received oral administration of a resveratrol solution at 10 mL/kg, while those in the RES-NLCs group received oral administration of resveratrol-loaded nanostructured lipid carriers at 10 mL/kg. The NA group and Blank group were orally administered an equal volume of 0.9% physiological saline and blank nanostructured lipid carriers, respectively. All mice were treated once daily by oral gavage for 28 consecutive days. At the end of the treatment period, the mice were euthanized by gradual-fill CO_2_ inhalation using 100% CO_2_ as the gas source, at a displacement rate of approximately 10–30% of the chamber volume per minute. Animals were exposed for approximately 5 min until movement and respiration ceased and pupils were fixed and dilated, followed by an additional 2–3 min observation period to confirm death. Systematic necropsy and sample collection were then performed for subsequent safety evaluation.

#### 2.5.2. Gross Anatomical Observation and Organ Coefficients

Body weight of each mouse was recorded daily, and signs of toxicity were observed, including but not limited to soft stools, diarrhea, piloerection, hematuria, depression, pallor, and nasal discharge. At the end of the experiment, mice were immediately dissected after euthanasia. Major organs (heart, liver, spleen, lungs, kidneys, and brain) were examined grossly for any abnormalities in appearance, size, and color. The wet weight of each organ was measured using an electronic balance, and organ coefficients (organ weight/body weight) were calculated.

#### 2.5.3. Body Composition and MRI Examination

A body composition analyzer was used to quantitatively determine fat volume, muscle volume, and total body water in each group of mice on day 0 and day 28. On day 28, all mice were also subjected to magnetic resonance imaging (MRI) scanning using a small animal MRI system. All mice were fasted for 12 h prior to the measurements and were kept under anesthesia during the analysis.

#### 2.5.4. Hematological and Serum Biochemical Analysis

At the experimental endpoint, mice blood samples were collected via the orbital venous plexus and kept at 4 °C. For hematological analysis, 20 μL of whole blood was mixed with 120 μL of diluent, followed by analysis using an automated hematology analyzer. For serum biochemical analysis, blood samples were allowed to clot by standing at room temperature under sterile conditions for 1 h, then centrifuged at 2700× *g* for 10 min. The supernatant (serum) was collected for biochemical analysis [22].

#### 2.5.5. Cardiac Ultrasound Examination

At the end of the experiment, cardiac function of the experimental animals in each group was systematically evaluated using a small animal ultrasound diagnostic system. Cardiac functional and structural parameters were obtained from M-mode and B-mode ultrasound images using the built-in analysis software. The main parameters assessed included heart rate (HR), stroke volume (SV), cardiac output (CO), ejection fraction (EF), left ventricular posterior wall thickness at systole (LVPW;s), and left ventricular posterior wall thickness at diastole (LVPW;d), among other cardiac functional and structural parameters. CO was calculated as SV × HR. EF, SV and LVPW were derived or measured automatically by the system software.

#### 2.5.6. Tissue HE Staining

At the end of the experiment, stomach, duodenum, jejunum, ileum, cecum, colon, and liver tissues were collected from mice and fixed in 4% paraformaldehyde. After paraffin embedding, 5-μm sections were prepared and subjected to hematoxylin and eosin (HE) staining for histopathological analysis [23].

### 2.6. Statistical Analysis

The main pharmacokinetic parameters of RES, including maximum plasma concentration (C_max_), time to reach Cmax (T_max_), elimination half-life (T_1/2_), area under the plasma concentration–time curve from time zero to the last sampling point (AUC_0–t_), and area under the curve extrapolated to infinity (AUC_0–∞_, used to assess relative bioavailability), were calculated from the mean plasma drug concentration–time data by non-compartmental analysis using WinNonlin 6.2 software (Certara, Radnor, PA, USA). Concentrations below the validated lower limit of quantification (LLOQ) were treated as BLQ and handled according to standard non-compartmental pharmacokinetic practice: pre-dose BLQ values were set to zero, intermediate BLQ values were treated as missing, and terminal BLQ values were excluded from terminal-phase estimation. Statistical analyses were performed using GraphPad Prism 8.0 software (GraphPad Software, San Diego, CA, USA). Data are presented as the mean ± standard deviation (SD). All raw data were tested for normality (Shapiro–Wilk test) and homogeneity of variances (Bartlett’s test). For comparisons among more than two groups, one-way analysis of variance (ANOVA) followed by Tukey’s multiple comparison test was used when parametric test assumptions were met. If the data did not meet parametric assumptions, appropriate non-parametric tests were applied. For comparisons between two groups, an unpaired two-tailed Student’s *t*-test was used when appropriate. The level of statistical significance was set at * *p* < 0.05, ** *p* < 0.01, *** *p* < 0.001 and **** *p* < 0.0001.

## 3. Results

### 3.1. Pharmacokinetic Analysis

**Figure 2:**
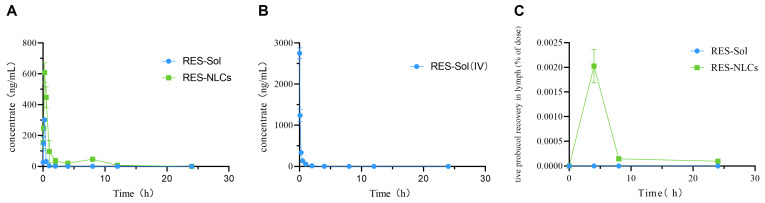
Pharmacokinetic profiles and lymphatic recovery of resveratrol following oral or intravenous administration in rats. (**A**) Plasma concentration–time curves of RES-Sol and RES-NLCs after oral administration. (**B**) Plasma concentration–time curve of RES-Sol after intravenous administration. (**C**) Time-dependent recovery of resveratrol in lymph, expressed as the percentage of the administered dose, after oral administration of RES-Sol and RES-NLCs. RES-Sol, resveratrol solution; RES-NLCs, resveratrol-loaded nanostructured lipid carriers; IV, intravenous.

The plasma concentration–time curve of RES is shown in Figure 2A,B. All plasma concentration data were analyzed by non-compartmental analysis using WinNonlin 6.2 software, and the resulting pharmacokinetic parameters are summarized in Table 1. The results showed that the peak concentration (C_max_) in the RES-Sol group was 263 ± 86.31 ng/mL, whereas that in the RES-NLCs group increased to 608 ± 63.96 ng/mL, representing an approximately 2.3-fold increase, with a highly statistically significant difference (*p* = 1.36 × 10^−5^). The time to reach Cmax (Tmax) was 15 min after administration in both the RES-Sol and RES-NLCs groups. Using the RES-Sol group as a reference, the area under the concentration–time curve (AUC_0–t_) of the RES-NLCs group was significantly increased, with a relative bioavailability approximately 7-fold higher (*p* = 7.42 × 10^−8^). When the intravenous (IV) group was used as a reference, the absolute bioavailability of the RES-NLCs group was 176%.

To directly assess drug transport through the intestinal lymphatic pathway, a mesenteric lymph duct–jugular vein assisted reflux model was applied in this study. The results are shown in Figure 2C, and the relevant parameters are presented in Table 2. Drug distribution was detectable in the lymph of the RES-NLCs group, whereas no drug concentration was detected in the lymph of the RES-Sol group throughout the entire sampling period.

### 3.2. In Vivo Safety Analysis

#### 3.2.1. Body Weight and Organ Coefficient Analysis

**Figure 3:**
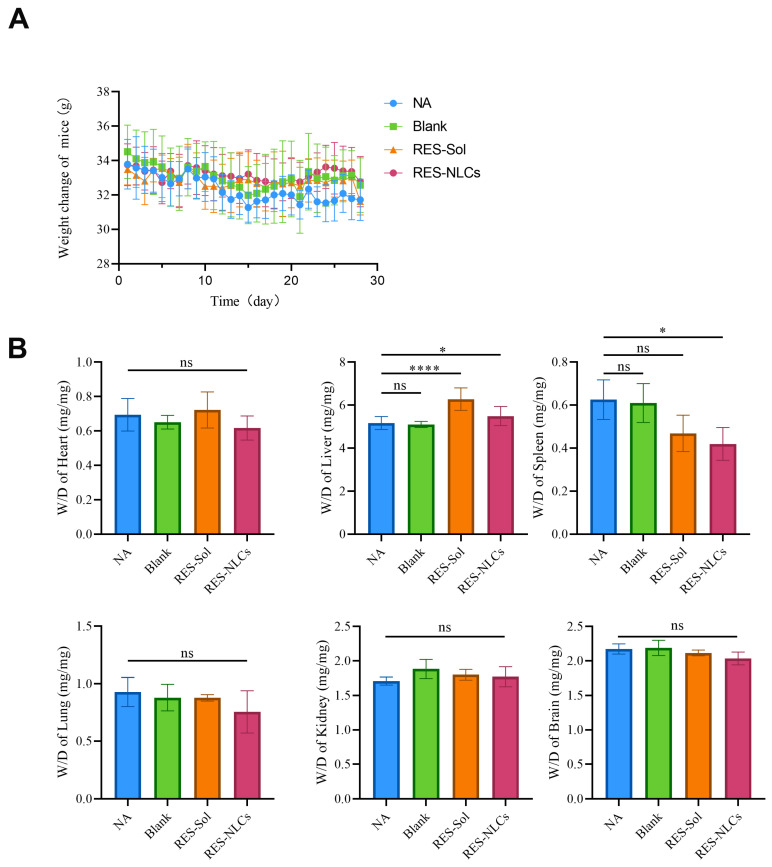
Body weight changes and organ coefficients of mice after 28 days of repeated oral administration. (**A**) Body weight changes in mice in the NA, Blank, RES-Sol, and RES-NLCs groups during the 28-day treatment period. (**B**) Organ coefficients of the heart, liver, spleen, lung, kidney, and brain in different groups of mice at the end of the experiment. Organ coefficients were calculated as wet organ weight/body weight. NA, normal control group; Blank, blank nanostructured lipid carrier group; RES-Sol, resveratrol solution group; RES-NLCs, resveratrol-loaded nanostructured lipid carrier group. Data are presented as mean ± SD. ns, not significant; * *p* < 0.05; **** *p* < 0.0001.

All mice in each group survived well without obvious clinical symptoms (Figure 3). Body weights in the NA and Blank groups showed a slight decrease, whereas those in the RES-Sol and RES-NLCs groups increased (Figure 3A). Organ coefficients of major organs (heart, liver, spleen, lungs, kidneys, and brain) were calculated. Compared with the NA group, the Blank group showed no significant difference in liver organ coefficient (*p* = 0.9915). The liver organ coefficient was significantly increased in the RES-Sol group (*p* = 0.0006) and also significantly increased in the RES-NLCs group (*p* = 0.0471). In contrast, the spleen organ coefficient was significantly decreased in the RES-NLCs group (*p* = 0.0201). No statistically significant differences were observed in the organ coefficients of the heart, lungs, kidneys, or brain among the groups (*p* > 0.05) (Figure 3B).

#### 3.2.2. Body Composition and MRI Findings

**Figure 4:**
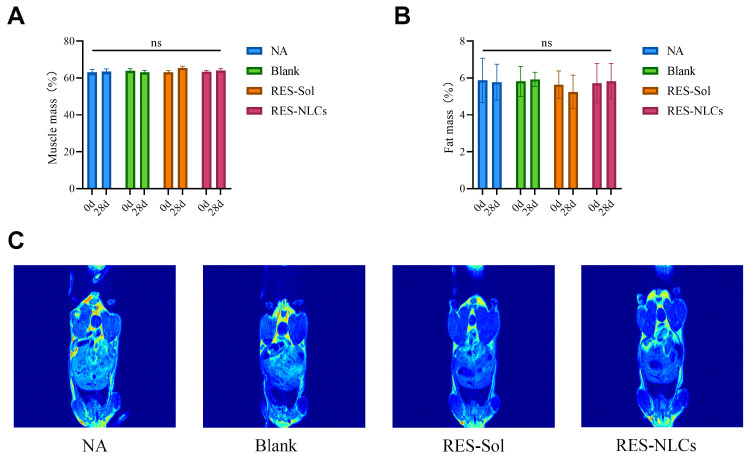
Body composition analysis of mice after 28 days of repeated oral administration. (**A**) Percentage of muscle mass in mice from the NA, Blank, RES-Sol, and RES-NLCs groups on day 0 and day 28. (**B**) Percentage of fat mass in mice from each group on day 0 and day 28. (**C**) Representative magnetic resonance imaging (MRI) images of mice from each group at the end of the experiment. NA, normal control group; Blank, blank nanostructured lipid carrier group; RES-Sol, resveratrol solution group; RES-NLCs, resveratrol-loaded nanostructured lipid carrier group. Data are presented as mean ± SD. ns, not significant.

The results of body composition analysis are shown in Figure 4A (muscle mass) and Figure 4B (fat mass). No significant changes in body composition were observed among the groups (*p* > 0.05). Furthermore, magnetic resonance imaging (MRI) (Figure 4C) revealed no obvious structural differences.

#### 3.2.3. Hematological Analysis

**Figure 5:**
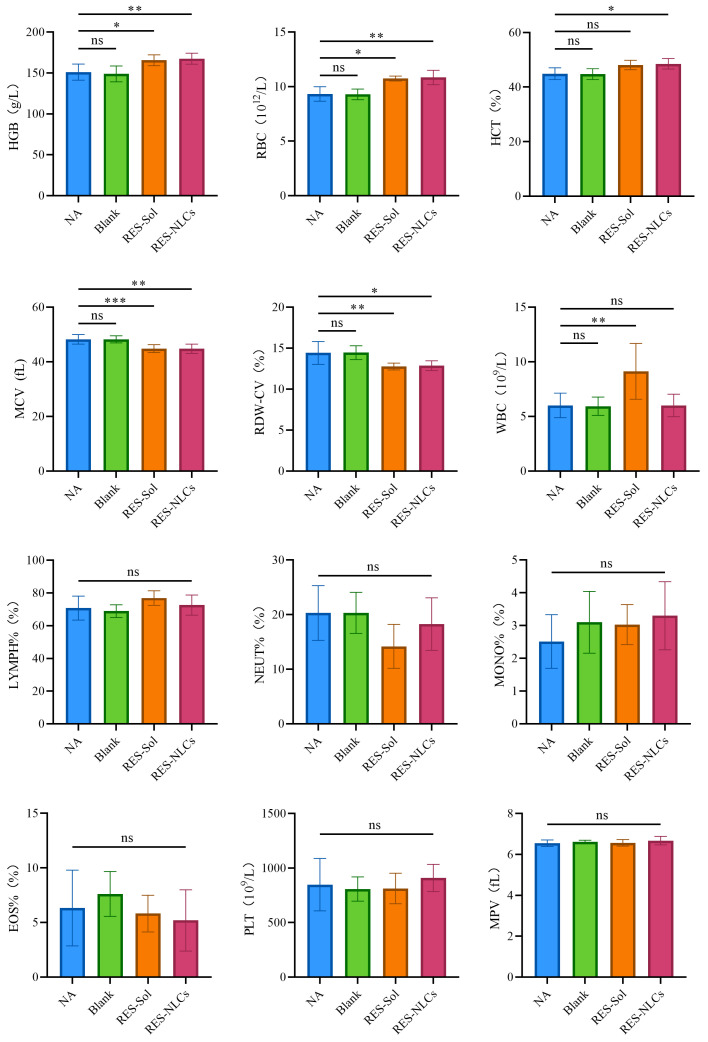
Hematological parameters of mice after 28 days of repeated oral administration. Hematological parameters were measured in mice from the NA, Blank, RES-Sol, and RES-NLCs groups at the end of the experiment, including hemoglobin concentration (HGB), red blood cell count (RBC), hematocrit (HCT), mean corpuscular volume (MCV), red blood cell distribution width coefficient of variation (RDW-CV), white blood cell count (WBC), lymphocyte percentage (LYMPH%), neutrophil percentage (NEUT%), monocyte percentage (MONO%), eosinophil percentage (EOS%), platelet count (PLT), and mean platelet volume (MPV). NA, normal control group; Blank, blank nanostructured lipid carrier group; RES-Sol, resveratrol solution group; RES-NLCs, resveratrol-loaded nanostructured lipid carrier group. Data are presented as mean ± SD. ns, not significant; * *p* < 0.05; ** *p* < 0.01; *** *p* < 0.001.

Hematological parameters are shown in Figure 5. No significant differences were observed between the Blank group and the NA group in any of the measured parameters. Compared with the NA group, the RES-Sol group exhibited significantly increased red blood cell (RBC) and white blood cell (WBC) counts (*p* = 0.0276 and *p* = 0.0068, respectively), whereas mean corpuscular volume (MCV) and red blood cell distribution width coefficient of variation (RDW-CV) were significantly decreased (*p* = 0.0008 and *p* = 0.0099, respectively). In the RES-NLCs group, RBC count was significantly increased (*p* = 0.0057), while MCV and RDW-CV were also significantly decreased (*p* = 0.0011 and *p* = 0.0131, respectively). No statistically significant differences were found in the other hematological parameters among the groups, including lymphocytes (Lymph), neutrophils (NEUT), monocytes (MONO), eosinophils (EOS), platelets (PLT), and mean platelet volume (MPV).

#### 3.2.4. Serum Biochemical Analysis

**Figure 6:**
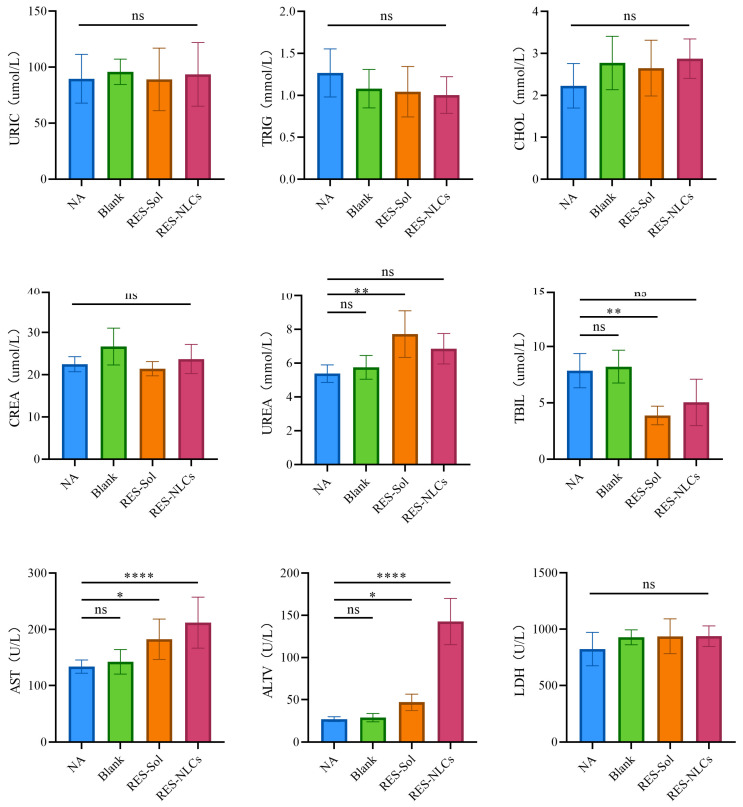
Serum biochemical parameters of mice after 28 days of repeated oral administration. Serum biochemical parameters were measured in mice from the NA, Blank, RES-Sol, and RES-NLCs groups at the end of the experiment, including uric acid (URIC), triglycerides (TRIG), total cholesterol (CHOL), creatinine (CREA), urea (UREA), total bilirubin (TBIL), aspartate aminotransferase (AST), alanine aminotransferase (ALT), and lactate dehydrogenase (LDH). NA, normal control group; Blank, blank nanostructured lipid carrier group; RES-Sol, resveratrol solution group; RES-NLCs, resveratrol-loaded nanostructured lipid carrier group. Data are presented as mean ± SD. ns, not significant; * *p* < 0.05; ** *p* < 0.01; **** *p* < 0.0001.

Serum biochemical parameters are shown in Figure 6. Compared with the NA group, the RES-Sol group showed a significant increase in urea (UREA) levels (*p* = 0.002), whereas the RES-NLCs group exhibited no significant change in UREA. No significant difference in creatinine (CREA) was observed between the RES-Sol group and the NA group. Similarly, the RES-NLCs group showed no significant change in CREA.

Regarding liver function-related indicators, compared with the NA group, the RES-NLCs group exhibited highly significant increases in aspartate aminotransferase (AST) and alanine aminotransferase (ALT) (*p* = 9 × 10^−5^ and *p* = 4.02 × 10^−7^, respectively). The RES-Sol group also showed significant increases in AST and ALT compared with the NA group (*p* = 0.0195 and *p* = 0.0162, respectively).

No statistically significant differences were observed in the remaining blood biochemical parameters among the groups (*p* > 0.05). Specifically, no significant differences were found in uric acid (URIC), triglycerides (TRIG), total cholesterol (CHOL), creatinine (CREA), and lactate dehydrogenase (LDH) across all groups.

#### 3.2.5. Analysis of Cardiac Function Indicators

**Figure 7:**
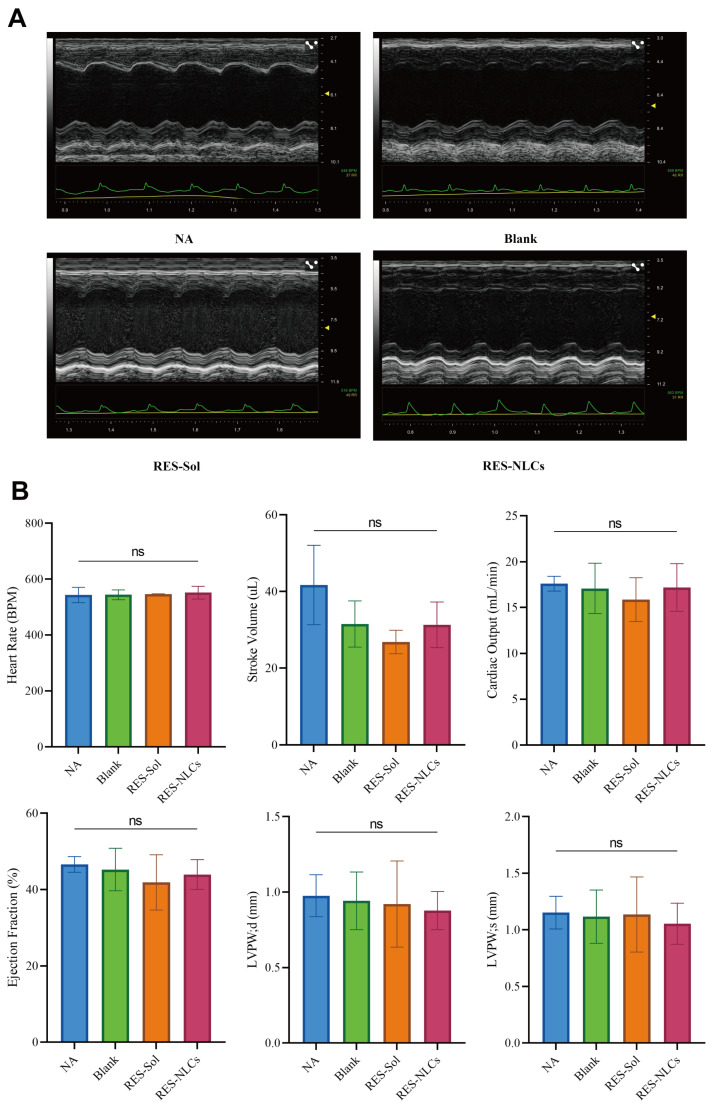
Cardiac ultrasound evaluation of mice after 28 days of repeated oral administration. (**A**) Representative M-mode echocardiographic images showing cardiac wall motion in mice from the NA, Blank, RES-Sol, and RES-NLCs groups. (**B**) Quantitative cardiac ultrasound parameters measured or calculated using the built-in analysis software, including heart rate (HR), stroke volume (SV), cardiac output (CO), ejection fraction (EF), left ventricular posterior wall thickness at diastole (LVPW;d), and left ventricular posterior wall thickness at systole (LVPW;s). NA, normal control group; Blank, blank nanostructured lipid carrier group; RES-Sol, resveratrol solution group; RES-NLCs, resveratrol-loaded nanostructured lipid carrier group. Data are presented as mean ± SD. ns, not significant.

After administration, cardiac ultrasound was performed on mice in each group, and the results are shown in Figure 7. There were no statistically significant differences in core cardiac function and structural parameters, including heart rate (HR), stroke volume (SV), cardiac output (CO), ejection fraction (EF), left ventricular posterior wall thickness at systole (LVPW;s), and left ventricular posterior wall thickness at diastole (LVPW;d), among all groups (*p* > 0.05).

#### 3.2.6. Histopathological Examination

**Figure 8:**
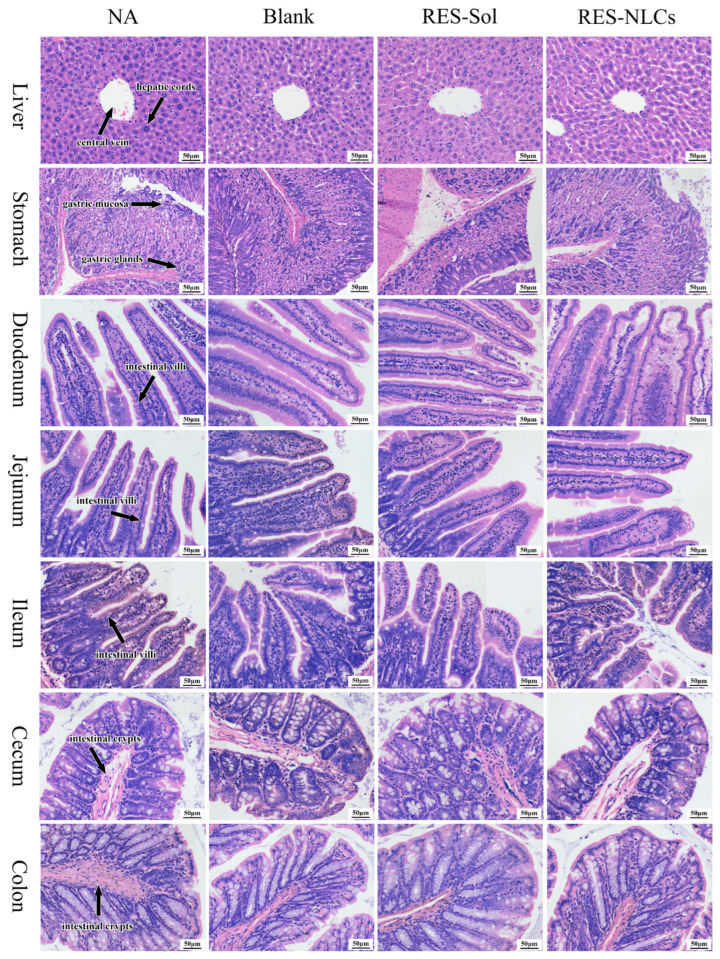
Histopathological examination of major organs and intestinal segments after 28 days of repeated oral administration. Representative hematoxylin and eosin (HE)-stained sections of the liver, stomach, duodenum, jejunum, ileum, cecum, and colon from mice in the NA, Blank, RES-Sol, and RES-NLCs groups. Arrows in the NA group indicate representative normal histological structures, including the hepatic central vein, hepatic cords, gastric mucosa, gastric glands, intestinal villi, and intestinal crypts. No obvious pathological lesions were observed in any group. NA, normal control group; Blank, blank nanostructured lipid carrier group; RES-Sol, resveratrol solution group; RES-NLCs, resveratrol-loaded nanostructured lipid carrier group. Scale bars = 50 μm.

HE staining sections of the mouse liver, stomach, duodenum, jejunum, ileum, cecum, and colon tissues were examined microscopically, and representative images are shown in Figure 8. No obvious pathological changes were observed in any group of animals, with intact tissue structures and normal cell morphology.

## 4. Discussion

In this study, RES-NLCs were developed to overcome the major limitation of resveratrol (RES) in veterinary applications—its low oral bioavailability. By integrating pharmacokinetic and systemic safety evaluations, this study showed that RES-NLCs improved the oral absorption efficiency of RES and were preliminarily tolerated under the present experimental conditions. These results provide useful preclinical data for the further development of RES-NLCs as a potential veterinary formulation.

Pharmacokinetic studies confirmed that RES-NLCs offer substantial advantages in improving oral absorption. Compared with RES-Sol, RES-NLCs increased the relative bioavailability by approximately 7-fold and exhibited an “absolute bioavailability exceeding 100%”—a phenomenon of super-bioavailability, suggesting that the absorption-enhancing mechanism may go beyond conventional solubility improvement. Our research group previously demonstrated, using the same lipid formulation, that this carrier system can mediate drug transport via the intestinal lymphatic pathway [16]. Accordingly, RES-NLCs likely deliver RES directly to the systemic circulation through a similar lymphatic-targeting mechanism, thereby effectively bypassing hepatic first-pass metabolism, which is considered the core reason for the marked improvement in bioavailability. A major finding of this study was the direct demonstration of intestinal lymphatic involvement in RES-NLCs absorption. Using a conscious rat mesenteric lymph duct-jugular vein shunt model, RES was consistently detected in the lymph of the RES-NLCs group, whereas it remained undetectable in the RES-Sol group. These results indicate that RES-NLCs can promote RES entry into systemic circulation through the intestinal lymphatic route, thereby providing direct mechanistic evidence for the observed enhancement in oral bioavailability.

The safety evaluation results indicated that at a dose of 10 mg/kg, no obvious toxic reactions were observed in any group of mice, suggesting preliminary tolerability of this lipid-based formulation under the present experimental conditions. First, the Blank group exhibited no significant toxicity, confirming that the nanocarrier material itself was well tolerated—an important prerequisite for further formulation development. Second, although the RES-NLCs group showed some physiological adaptive changes associated with increased drug exposure (e.g., elevated AST and ALT activities reflecting increased hepatic metabolic load or potential hepatic stress, as well as alterations in RBC, MCV and other parameters indicative of erythrocyte system regulation), histopathological examination of the liver and various intestinal segments revealed no obvious structural damage. These results suggest that the observed biochemical and hematological fluctuations were not accompanied by overt histopathological injury; however, the significant elevations of AST and ALT should not be overlooked and require further mechanistic and long-term safety investigations. Moreover, cardiac function parameters (heart rate, stroke volume, ejection fraction, cardiac output, etc.) showed no statistically significant differences among all groups, further supporting the cardiac safety of the formulation. Notably, compared with the RES-Sol group, the RES-NLCs group did not exhibit an increased renal burden (as indicated by urea, UREA) and even showed a tendency toward renal protection, possibly related to reduced direct renal exposure due to lymphatic absorption.

The LC-MS/MS method used in this study was adapted from previously reported methods for RES quantification and evaluated using linearity assessment, batch-specific calibration curves, and representative chromatograms. However, it was not fully validated according to regulatory bioanalytical guidelines. Therefore, this method should be regarded as suitable for comparative preclinical pharmacokinetic analysis under the present experimental conditions. If this LC-MS/MS method is to be used in the future for regulatory drug evaluation, routine bioanalysis, or standardized analytical application, full bioanalytical method validation will be required, including accuracy, precision, extraction recovery, matrix effect, carryover, dilution integrity, multiple stability assessments, and cross-validation. We plan to conduct this comprehensive validation as a future independent study. In addition, this study has several limitations, including the relatively small sample size, the use of only female mice in the safety evaluation, the short observation period, the lack of oxidative stress parameters, and the absence of validation in target livestock species.

## 5. Conclusions

In summary, RES-NLCs improved the oral exposure of resveratrol in rats, and the mesenteric lymph collection model provided direct evidence that intestinal lymphatic transport contributed to this enhanced absorption. In the 28-day repeated-dose safety evaluation, RES-NLCs caused no overt histopathological damage in major organs, suggesting preliminary tolerability of this lipid-based formulation. These findings indicate that NLCs may represent a useful oral delivery strategy for poorly soluble compounds and provide preclinical data for the further development of RES-based veterinary formulations. Overall, these findings should be interpreted as preclinical evidence obtained from rodent models, and further studies in target livestock species are required to confirm their practical veterinary relevance. Future studies should include mechanistic safety evaluations, comprehensive analytical method validation and cross-validation, long-term and reproductive toxicity assessments, residue depletion studies, and validation in target animals such as pigs and poultry.

## Figures and Tables

**Table 1:** Pharmacokinetic parameters.

Parameters	RES-Sol (Oral)	RES-NLCs (Oral)	RES-Sol (IV)
Cmax (ng/mL)	263 ± 86.31	608 ± 63.96 ****	2751.67 ± 130.14 ****
Tmax (h)	0.25	0.25	0.033 **
T1/2 (h)	3.30 ± 1.54	2.83 ± 1.12	1.20 ± 0.55
AUC_0–t_ (ng·h/mL)	95.31 ± 30.35	708.48 ± 103.98 ****	401.10 ± 45.87 ****
AUC_0–∞_ (ng·h/mL)	94 ± 29.37	741.43 ± 97.90 ****	402.77 ± 46.14 ****

Note: Data are presented as mean ± SD. ** and **** indicate *p* < 0.01 and *p* < 0.0001, respectively, compared with the RES-Sol (Oral) group.

**Table 2:** Dose percentage parameters in rat lymph fluid.

Parameters	Liquid Collection Time, h	RES-Sol	RES-NLCs
Concentration, ng·mL^−1^	0–4	0	441.67 ± 27.09
	4–8	0	32.77 ± 1.25
	8–24	0	7.25 ± 0.85
V, mL	0–4	4.12 ± 0.51	4.61 ± 0.74
	4–8	4.58 ± 0.69	4.43 ± 0.63
	8–24	12.86 ± 0.99	13.24 ± 1.05
Dose percentage, %	0–4	0	0.034 ± 0.005
	4–8	0	0.002 ± 0.0003
	8–24	0	0.002 ± 0.0002

## Data Availability

The raw data supporting the conclusions of this article will be made available by the authors on request.

## Supplementary Materials

The following supporting information can be downloaded at: https://www.mdpi.com/article/10.3390/ani16152337/s1, File S1: LC-MS Standard Curves and Chromatograms.

## Abbreviations

The following abbreviations are used in this manuscript:

RES	resveratrol
RES-NLCs	resveratrol-loaded nanostructured lipid carriers
NLCs	nanostructured lipid carriers
RES-Sol	resveratrol solution
MRM	multiple reaction monitoring
HR	heart rate
SV	stroke volume
CO	cardiac output
EF	ejection fraction
LVPW	left ventricular posterior wall thickness at systole
URIC	uric acid
TRIG	triglycerides
CREA	creatinine
LDH	lactate dehydrogenase
Lymph	lymphocytes
NEUT	neutrophils
MONO	monocytes
EOS	eosinophils
PLT	platelets
MPV	mean platelet volume
WBC	white blood cell
RBC	red blood cell
MCV	mean corpuscular volume
RDW-CV	red blood cell distribution width coefficient of variation
AST	aspartate aminotransferase
ALT	alanine aminotransferase
